# Overexpression of *PtrPIP2:4* Accelerates Adventitious Root Emergence, Promotes Adventitious Root Elongation, and Increases Lateral Root Number in Poplar

**DOI:** 10.3390/plants15121844

**Published:** 2026-06-15

**Authors:** Hao Cheng, Ge Zhao, Wenli Li, Yuxiang Cheng

**Affiliations:** 1 State Key Laboratory of Tree Genetics and Breeding, Northeast Forestry University, Harbin 150040, China; chenghaolucky@126.com (H.C.); gracezhao0929@126.com (G.Z.); 17658255362@163.com (W.L.); 2 College of Life Sciences, Northeast Forestry University, Harbin 150040, China

**Keywords:** *Populus trichocarpa*, *PtrPIP2:4*, adventitious root, auxin-related gene, transcriptome

## Abstract

Plasma membrane intrinsic proteins (PIPs), a subfamily of aquaporins (AQPs), play critical roles in various physiological processes in plants, including the transport of water and CO_2_, regulation of stomatal movement, absorption of neutral molecules and nutrients, and H_2_O_2_ signaling. Nevertheless, the functions of *PIP* aquaporins in adventitious root formation in trees are still poorly understood. *PtrPIP2:4* is specifically expressed in roots, and PtrPIP2:4 fused with GFP localizes to the plasma membrane. Overexpression of *PtrPIP2:4* significantly accelerated adventitious root induction in poplar. Stem cuttings from overexpression lines exhibited more rapid rooting compared to wild-type (WT) plants, although the total number of adventitious roots did not differ significantly. Additionally, the number of lateral roots was markedly increased in *PtrPIP2:4* overexpression lines. Comparative transcriptome analysis identified 4204 differentially expressed genes (DEGs) between WT and *PtrPIP2:4* overexpression plants. Transcriptomic analysis revealed that genes associated with auxin-related and flavonoid biosynthesis were significantly enriched. RT-qPCR results showed that the transcription levels of nine auxin-related genes (i.e., *PtrARF*, *PtrIAA*, *PtrGH3* and *PtrPIN*) were significantly upregulated, while the transcription levels of five flavonoid synthesis genes (i.e., *PtrDFR*, *PtrANS*, *PtrANR* and *PtrLAR*) were also significantly upregulated. Previous studies have implicated these genes in adventitious root formation. Collectively, these findings reveal that *PtrPIP2:4* accelerates adventitious root emergence, promotes adventitious root elongation, and increases lateral root number while the total number of adventitious roots exhibited no significant difference in poplar, suggesting its potential utility in improving tree propagation and breeding strategies.

## 1. Introduction

Adventitious roots frequently grow from cells near vascular tissues. In Arabidopsis, vascular tissues of petioles or stem cuttings undergo significant cell division to create the microcallus, indicating adventitious root founder cells [1,2]. In woody species, adventitious root primordia develop from cells located between vascular bundles and the starch sheath layer. In cuttings, adventitious roots form from stem pericycle-like tissue close to vascular bundles or from differentiated calli following injury [3]. Numerous endogenous and exogenous factors influence adventitious root formation, including hormones (particularly auxin), temperature, and flavonoids [4,5,6]. In the auxin-dependent pathway, PIN-FORMED1 (*PIN1*) and *PIN2* regulate polar auxin transport to control AR formation in Arabidopsis hypocotyls [7], while RNA interference of *OsPIN1* in rice inhibits AR emergence and elongation [8]. Another important gene involved in auxin signal transduction is AUXIN RESPONSE FACTOR 17 (*ARF17*), which is a target of *microRNA160* and inhibits AR development [9]. Despite several achievements in the subject, more critical genetic factors and their action mechanisms governing adventitious root formation remain to be uncovered, particularly in trees.

Plasma membrane intrinsic proteins (PIPs) are a subfamily of aquaporins (AQPs) in plants [10]. PIPs have PIP1 and PIP2 subtypes, with conservative folding and membrane topology structures composed of six transmembrane helices. PIP1 has a long N-terminus and a short C-terminus, while PIP2 is the opposite. *PIPs* and their members often form tetramers of the same or different species [11], promoting the transport of small molecules such as water. Plant *PIPs* have been implicated in several development processes, including cell expansion, organ movement, and elongation [12,13,14]. For example, knockdown of expression of *GhPIP2* genes in cotton by RNA interference hinders fiber elongation [15]; silencing of *Rh-PIP2:1* resulted in petals with significantly decreased length and width in Rose [16]; and in tulip (*Tulipa* spp.), temperature-dependent changes in a putative plasma membrane-associated AQP activity were associated with petal opening and closing [17]. In addition, *PIPs* play important roles in plant responses to abiotic stress. Overexpression of TaPIP1A from wheat improves drought and salt tolerance in *Arabidopsis* via interaction with TaPIP:3 and regulation by TaWRKY71 [18]. Overexpression of the *SmPIP1:3* gene in tobacco confers combined tolerances to drought, salt, cold, and heat stress [19]. Most existing studies on poplar *PIPs* concentrate on drought tolerance, embolism repair, and photosynthetic regulation [20,21,22]. By contrast, little is known about the roles of *PIP* genes in adventitious root formation of woody plants.

In this study, we examined the expression patterns of all *PIP* genes in *P. trichocarpa*, and found that *PtrPIP2:4* was highly and specifically expressed in roots. We further found that constitutive overexpression of *PtrPIP2:4* accelerates adventitious root emergence, promotes adventitious root elongation, and increases lateral root number in *P. trichocarpa*. Adventitious roots appeared in transgenic stem cuttings two days earlier than in WT. *PtrPIP2:4* overexpression lines had substantially longer adventitious roots than WT, as well as a much higher lateral root number. The transcriptome data revealed that auxin-related genes and flavonoid biosynthesis genes were highly increased in *PtrPIP2:4*-overexpressing plants, indicating that *PtrPIP2:4* plays a role in adventitious root emergence, adventitious root elongation, and lateral root formation.

## 2. Results

### 2.1. Identification of PtrPIP Genes in P. trichocarpa

**Figure 1:**
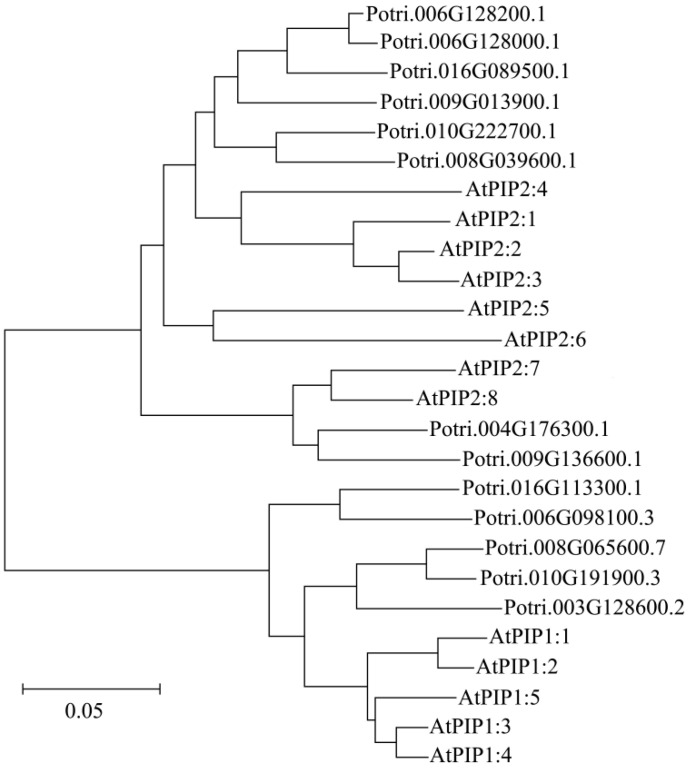
Phylogenetic analysis of *PIP* genes from *A. thaliana* and *P. trichocarpa*. A total of 13 PtrPIPs and 13 AtPIPs were aligned with Clustal W, and the phylogenic tree was constructed using MEGA 5.0’s neighbor-joining method with 1000 bootstrap replications. All *PIP* genes were classified into two clusters: *PIP1* and *PIP2*. The branch lengths of the phylogenetic tree denote genetic distance; the scale is 0.05.

To find the *PtrPIP* gene family in *P. trichocarpa*, we searched its genome for the Pfam thaumatin domain (PF00230) and the reported Arabidopsis AtPIPs sequences. As a result, 13 *PtrPIP* genes were identified in *P. trichocarpa* (Table S1). The amino acid multiple sequence alignment of 13 *PtrPIP* candidate genes in *P. trichocarpa* revealed that the PIP has six conserved transmembrane domains and two NAP conserved motifs (Figure S1). In addition, the first transmembrane domain has a conserved AEF motif (Figure S1). Phylogenetic analyses were then performed using the protein sequences of the PIP families from *A. thaliana* and *P. trichocarpa*. The results showed that the 13 PtrPIPs in *P. trichocarpa* could be classified into two distinct subgroups: PtrPIP1 (five members) and PtrPIP2 (eight members) (Figure 1).

Thirteen *PtrPIP* genes in all were scattered and asymmetrically distributed among seven chromosomes (Figure S2). There was one *PtrPIP* gene on chromosomes 3 and 4, and two on each of chromosomes 8, 9, 10, and 16. With three *PtrPIPs*, chromosome 4 has the most members. All of the *PtrPIP1* and *PtrPIP2* genes were designated *PtrPIP1:1* to *PtrPIP1:5* and *PtrPIP2:1* to *PtrPIP2:8*, respectively, based on the gene arrangement on the chromosomes (Figure S1). The ProtParam website (https://web.expasy.org/protparam/; accessed on 12 April 2025) was used to carry out physicochemical analysis of these identified genes. The *PtrPIP* family proteins ranged in molecular weight from 29.57 to 38.67 kD and in length from 280 to 375 amino acids (Table S2). Subcellular localization prediction indicated that all PtrPIPs were localized in the plasma membrane (Table S2).

### 2.2. PtrPIP2:4 Gene Is Dominantly Expressed in Roots

**Figure 2:**
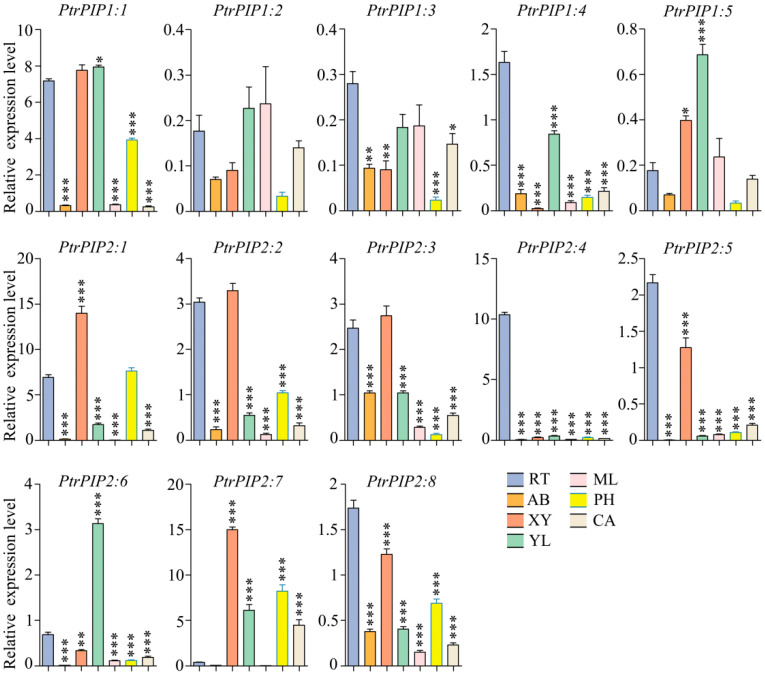
Expression profiles of *PtrPIP* genes in different tissues of *P. trichocarpa* using RT-qPCR. The tissues examined were roots (RT), apical buds (AB), xylem (XY), young leaves (YL), mature leaves (ML), phloem (PH), and rich cambium (CA). The expression of *PtrActin2* served as an internal control, and the values are the mean ± SDs of three technical replicates. The *t*-test revealed a statistically significant difference between RT and other tissues. Differential expression profiles were determined by |fold change| > 2, *p* < 0.05. Asterisks indicate a significant difference from WT using Student’s *t*-test (* *p* < 0.05; ** *p* < 0.01; *** *p* < 0.001).

To gain insight into the functions of *PtrPIP* genes, we examined their transcription levels across multiple tissues, including mature leaf, young leaf, root, xylem, phloem, cambium, and apical bud by RT-qPCR (Figure 2). For the five *PtrPIP1* genes, *PtrPIP1:1* was primarily expressed in roots, xylem, young leaves, and phloem, with its expression level in roots reaching 7 times higher than *PtrActin2* (as a control gene); however, its expression levels were lower in mature leaves, cambium and apical buds. The expression levels of *PIP1:2*, *PIP1:3*, and *PIP1:5* were lower than actin in all tissues. *PIP1:4* was preferentially expressed in roots and xylem, with the highest expression in roots reaching 1.7 times that of *PtrActin2*.

For the eight *PtrPIP2* genes (Figure 2), *PtrPIP2:1* was preferentially expressed in the root, xylem and phloem, with a 14-fold increase in xylem expression over *PtrActin2*. *PtrPIP2:2* and *PtrPIP2:3* showed similar expression profiles, particularly in root and xylem. *PtrPIP2:5* was highly expressed in roots and xylem, but *PtrPIP2:6* was only expressed in early leaves. *PtrPIP2:7* was highly expressed in early leaves, cambium, phloem, and xylem, while *PtrPIP2:8* was strongly expressed in roots, phloem, and xylem. It is worth mentioning that *PtrPIP2:4* demonstrated a distinct and high expression level in the root, up to 11 times that of *PtrActin2*. It implies that *PtrPIP2:4* may play a role in *P. trichocarpa* root development.

### 2.3. PtrPIP2:4-GFP Localizes to the Plasma Membrane

**Figure 3:**
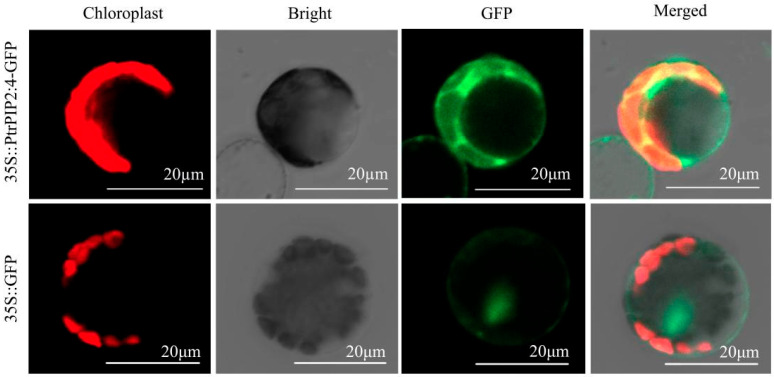
Subcellular localization of PtrPIP2:4-GFP in protoplasts. Fluorescence pictures were taken from *P. trichocarpa* leaf protoplasts using laser confocal microscopy. PtrPIP2;4-GFP fusion protein (green fluorescence) localizes exclusively to the plasma membrane in *Populus trichocarpa* protoplasts, whereas 35S::GFP serves as a negative control. Chloroplast autofluorescence is shown in red. Scale bars = 20 μm.

To understand the role of *PtrPIP* genes in root formation, we selected *PtrPIP2:4*, a gene with specific high expression in roots, for further functional research. First, we investigated the localization of PtrPIP2:4 in vivo. The recombinant construct of the PtrPIP2:4 fused to the green fluorescent protein (GFP) reporter gene driven by the cauliflower mosaic virus (CaMV) 35S promoter was transformed in *P. trichocarpa* leaf protoplasts. As shown in Figure 3, the PtrPIP2:4-GFP fusion protein was found to accumulate exclusively in the plasma membrane while the control protein GFP was distributed throughout the whole cell. The findings suggest that PtrPIP2:4 localizes in the plasma membrane.

### 2.4. Overexpression of PtrPIP2:4 Accelerates Adventitious Root Emergence, Promotes Adventitious Root Elongation, and Increases Lateral Root Number in P. trichocarpa

**Figure 4:**
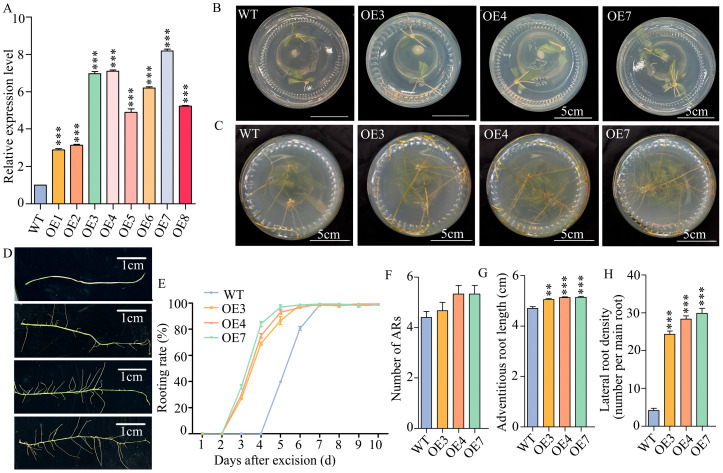
Overexpression of *PtrPIP2:4* in poplar accelerates adventitious root emergence, promotes adventitious root elongation, and increases lateral root number. (**A**) RT-qPCR analysis of PtrPIP2:4 transcription levels in its overexpression lines (OE1-OE8) and wild-type (WT) plants. (**B**) Phenotypes of the OE and WT rooting on the WPM media. Bar: 5 cm. (**C**) Phenotypes of the OE and WT rooting on the WPM media for 30 days. Bar: 5 cm. (**D**) Phenotypes of OE and WT lateral roots on the thirtieth day. Bar: 1 cm. (**E**) Statistics of rooting rates of OE and WT plants in the 10-day period. (**F**,**G**) The number (**F**) and length (**G**) of adventitious roots per OE and WT cutting for 30 days. (**H**) The number of lateral roots per OE and WT adventitious root for 30 days. In (**E**–**H**), the values are means ± SDs (*n* = 30). Asterisks indicate a significant difference from WT using Student’s *t*-test (** *p* < 0.01 and *** *p* < 0.001).

To explore the biological function of *PtrPIP2;4*, we transformed *Populus trichocarpa* with its overexpression vector and obtained eight independent 35S::*PtrPIP2;4* overexpression (OE) lines. Following RT-qPCR detection, the *PtrPIP2:4* transcript level increased by 3 times in OE1 and OE2 lines, 5–6 times in OE5, OE6, and OE8 lines, and 7–8 times in OE3, OE4, and OE7 lines, respectively (Figure 4A). The results indicate that these transgenic lines have overexpressed *PtrPIP2:4* gene in poplar. The three lines OE3, OE4, and OE7 with high expression of *PtrPIP2:4* were used for subsequent functional analysis.

To examine the roles of *PtrPIP2:4* in root formation, the basal ends of stem cuttings from *PtrPIP2:4*-OE lines and WT plants were cultivated on WPM media to investigate rooting phenotypes. Under the same conditions, adventitious roots rapidly developed in the cuttings of three *PtrPIP2:4*-OE lines, emerging two days earlier than the WT (Figure 4B,C). After 30 days of culture on WPM media, we found no significant difference in the average number of adventitious roots between *PtrPIP2:4*-OE lines and WT cuts (Figure 4E). In contrast, adventitious roots from the *PtrPIP2:4*-OE cuttings were much longer than those from the WT’s (Figure 4F). Furthermore, the average number of lateral roots from the *PtrPIP2:4*-OE cuttings was significantly higher than that of the WT; each WT adventitious root has approximately four lateral roots, whereas each *PtrPIP2:4*-OE adventitious root has more than 24 lateral roots (Figure 4D,G). These findings show that overexpression of *PtrPIP2:4* accelerates adventitious root emergence, promotes adventitious root elongation, and increases lateral root number in *P. trichocarpa*, but has no effect on the final total number of adventitious roots.

### 2.5. Transcriptome Analysis of the PtrPIP2:4 Overexpression Plants

**Figure 5:**
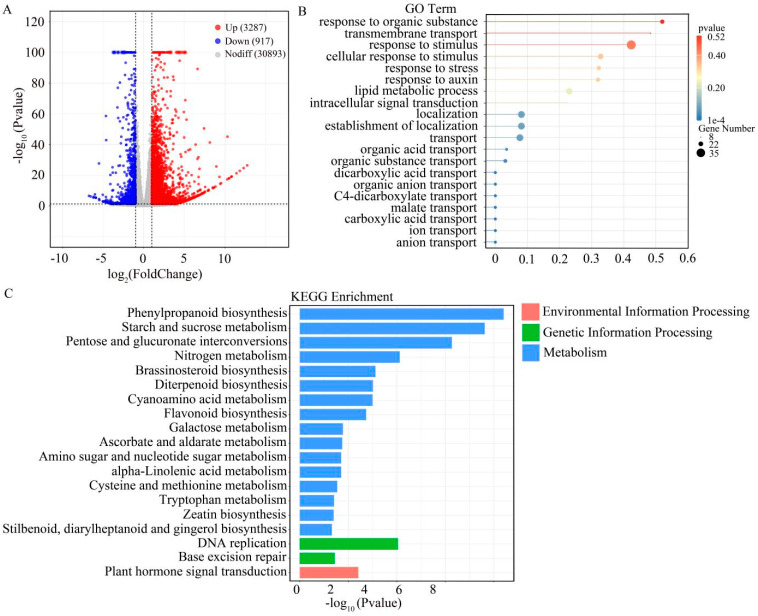
Transcriptome analysis of roots in *PtrPIP2:4* overexpression plants. (**A**) Volcano plot of DEGs between overexpression line and the control. (**B**) GO enrichment analysis of DEGs between overexpression line and the control. (**C**) KEGG enrichment analysis of DEG overexpression line and the control.

To investigate the regulatory network underlying *PtrPIP2:4* overexpression in promoting adventitious root formation, transcriptome sequencing was performed on roots of WT and *PtrPIP2:4*-OE plants, with three biological replicates per group. The DESeq2 approach identified 4204 differentially expressed genes (DEGs) based on |log_2_ fold change| > 1 and FDR-adjusted *p *< 0.05 (Table S3). The volcano plot of these DEGs revealed significant upregulation of 3287 genes and downregulation of 917 genes in the roots of *PtrPIP2:4*-OE plants (Figure 5A).

To classify the functions of these differentially expressed genes, we performed Gene Ontology (GO) enrichment analysis, and they were separated into three major ontologies: biological process, cellular component, and molecular function. In the biological process category, DEGs were significantly enriched in GO terms including response to organic substance, transmembrane transport, cellular response to stimuli, response to stress, response to auxin, and lipid metabolic process, etc. (Figure 5B). Furthermore, KEGG pathway analysis revealed that the DEGs were significantly enriched in phenylpropanoid biosynthesis, starch and sucrose metabolism, pentose and glucuronate interconversions, nitrogen metabolism, brassinosteroid biosynthesis, diterpenoid biosynthesis, cyanoamino acid metabolism, and flavonoid biosynthesis, etc. (Figure 5C). Collectively, the GO and KEGG enrichment analyses indicated that DEGs from *PtrPIP2:4*-OE plants were significantly enriched in diverse pathways including auxin response and flavonoid biosynthesis.

### 2.6. Overexpression of PtrPIP2:4 in Poplar Affects the Expression of Auxin-Related and Flavonoid Synthesis Genes

**Figure 6:**
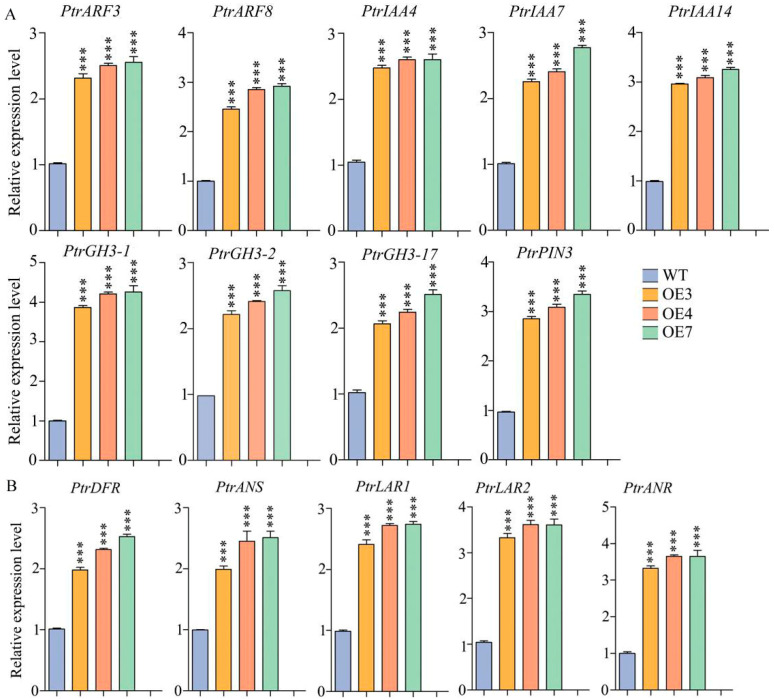
Expression levels of auxin and flavonoid-relate genes of *PtrPIP2:4*-OE and WT. (**A**) Expression levels of 9 auxin-relate genes of *PtrPIP2:4*-OE and WT. (**B**) Expression levels of 5 auxin-relate genes of *PtrPIP2:4*-OE and WT. The root of WT and *PtrPIP2:4*-OE plants was used to perform the RT-qPCR. Error bars represent ± SDs from three biological replicates, with three *P. trichocarpa* plants per genotype in each replicate (*** *p* < 0.001, Student’s *t*-test).

Previous studies indicate that auxin biosynthesis, polar transport, and signal transduction are crucial for adventitious root formation [23,24,25]. Compared to the WT, nine auxin-related genes showed significant transcriptional changes in *PtrPIP2:4* overexpression plants (Figure 6A). These genes belong to the *GH*, *IAA*, *ARF*, and *PIN* families. Specifically, *PtrGH3-1*, *PtrGH3-2*, and *PtrGH3-17* increased by 4.2-, 2.4-, and 2.6-fold; *PtrIAA4*, *PtrIAA7*, and *PtrIAA14* by 2.6-, 2.8-, and 3.2-fold; *PtrARF3* and *PtrARF8* by 2.6- and 3.0-fold; and the auxin transporter *PtrPIN3* by 3.3-fold. These results indicate that overexpression of *PtrPIP2:4* may regulate adventitious root formation by affecting the transcription of auxin-related genes, although the underlying mechanism remains to be further verified.

We found that transcript levels of several flavonoid biosynthesis genes were altered in *PtrPIP2:4* overexpression plants. These genes encode enzymes involved in the late stages of flavonoid production, including dihydroflavonol 4-reductase (*DFR*), anthocyanidin synthase (*ANS*), anthocyanidin reductase (*ANR*), and leucoanthocyanidin reductase (*LAR*). Compared to the WT, *PtrLAR1* and *PtrLAR2* transcripts increased by 2.7- and 3.6-fold, respectively, while *PtrANR* increased by 3.6-fold. Additionally, *PtrDFR* and *PtrANS*, which catalyze anthocyanin synthesis, were upregulated by 2.5- and 2.4-fold, respectively (Figure 6B). These findings indicate that overexpression of *PtrPIP2:4* may enhance the transcription of late-stage flavonoid biosynthesis genes in poplar.

## 3. Discussion

Adventitious root formation is a core process governing vegetative propagation efficiency and stress adaptability in woody plants. Yet the regulatory mechanism underlying tree root development mediated by *PIP2* remains largely uncharacterized. Previous studies in *Arabidopsis* reported that *PIP2:1* and *PIP2:5* modulate lateral root emergence and root growth under abiotic stresses [26,27]. Our study extends this understanding by identifying a root-specific *PtrPIP2:4*, which directly promotes adventitious root emergence, elongation, and lateral root formation. Notably, overexpression of *PtrPIP2:4* advanced adventitious root emergence by 2 days, significantly promoted root elongation and increased lateral root density, but did not alter the final total number of adventitious roots, indicating a specialized role of *PtrPIP2:4* in regulating adventitious root emergence, elongation, and lateral root formation in woody plants. In addition, combined transcriptomic analysis further revealed that the promotion of adventitious root development by *PtrPIP2:4* is correlated with upregulated expression of genes involved in auxin signaling and flavonoid biosynthesis pathways. These findings establish a novel functional link between *PIP2* and adventitious root emergence, elongation, and lateral root formation in woody species, providing new genetic targets for tree breeding aimed at improving propagation efficiency.

Auxin biosynthesis, transport, reversible conjugation and irreversible catabolism make up a set of complex mechanisms that jointly control the auxin distribution patterns that regulate plant root developmental processes. The ectopic expression of *OsGH3.2* in rice leads to production of few crown roots and root hairs [28]. Similarly, the *osgh3.13* mutant reportedly has fewer lateral and adventitious roots than the wild type [29]. The *atpin3* mutant significantly reduced the density of lateral roots [30]. Moreover, rice *osiaa3* mutants are insensitive to auxin and gravitropic stimuli, with fewer crown roots than in normal rice plants [31]. The root elongation zones of *osarf12* and *osarf12/25* mutants are significantly shorter than those of WT plants, probably because of a decrease in auxin synthesis and transport [32,33]. In this study, transcriptomic analysis showed that overexpression of *PtrPIP2:4* leads to upregulation of transcription levels of genes involved in auxin synthesis, auxin transport, and auxin reversible conjugation, including *GH3*, *IAA*, *ARF*, and *PIN* family members. Plant *PIPs* act as functional channels for H_2_O_2_ transport across cell membranes in all living cells. The influx of H_2_O_2_, which results in a transient rise in cellular H_2_O_2_ levels, can mediate auxin signaling and metabolism. For instance, H_2_O_2_ regulates the auxin gradients by the modulation of intercellular transport to regulate plant root apical meristem activity [34]. hydrogen peroxide (H_2_O_2_) triggers cellular mitogen activated protein kinase (MAPK) pathways and repressauxin-dependent signaling [35]. However, whether *PtrPIP2:4* regulates auxin through H_2_O_2_ transport to accelerate adventitious root emergence, elongation, and lateral root formation, still needs further verification.

Hydrogen peroxide affects not just the metabolism and signaling of auxin in cells, but also the biosynthesis of flavonoids. For example, in *Arabidopsis*, exogenous application of hydrogen peroxide can activate the expression of *ZAT* and positively regulate the biosynthesis of flavonoids [36]. *PuRBOHF*-dependent H_2_O_2_ in melatonin-induced anthocyanin accumulation in pears was shown by [37]. Flavonoids have also been shown to regulate root growth in herbaceous plants [38,39]. Tan et al. [40] determined that GA promotes root growth by directly inhibiting flavonol biosynthesis. Xu et al. [41] found that the flavonoid-induced transcriptional alterations to genes involved in auxin signaling inhibit cell elongation in the elongation zone of rice roots. Maloney et al. [42] proved that flavonols modulate auxin transport in tomato to positively and negatively affect lateral root formation and root hair formation. Overexpressing *GbDFR6* in tobacco can significantly increase the length of the primary root but reduces the number of lateral roots [43]. In this study, key structural genes in the late stage of flavonoid biosynthesis including *DFR*, *ANS*, *ANR*, and *LAR* were significantly upregulated in *PtrPIP2:4* overexpressing plants. This finding may uncovers a novel crosstalk between *PIP2*-mediated H_2_O_2_ transport to regulate flavonoid metabolism in tree adventitious root emergence, elongation, and lateral root formation, and the molecular mechanism still needs further exploration.

Transcriptomic data revealed that *PtrPIP2:4* overexpression is closely correlated with the upregulation of auxin-related genes and flavonoid biosynthesis pathways, which may jointly contribute to the phenotype. However, there is still a lack of physiological and genetic evidence to determine whether *PtrPIP2:4* accelerates adventitious root emergence, promotes root elongation, and increases lateral root number by altering auxin synthesis, signal transduction, and flavonoid synthesis. Therefore, in the future, we will measure the content of auxin and flavonoids in the roots of transgenic and wild-type plants. In addition, we will investigate whether exogenous application of auxin and flavonoid inhibitors can reverse the phenotype of overexpressing plants.

Furthermore, *PtrPIP2s* act as key membrane channels for water and small molecules such as H_2_O_2_. In the current version, we mainly focused on phenotypic observation, transcriptomic changes and downstream gene expression, but lacked direct physiological measurements of water transport and hydraulic properties, which makes the connection between its aquaporin property and rooting regulation insufficiently solid. Whether *PtrPIP2:4* accelerating adventitious root emergence, promoting root elongation, and increasing lateral root number relies on water or H_2_O_2_ transport activity needs further investigation. Future work will focus on measuring root hydraulic conductivity and substrate transport activity to dissect the plasma membrane intrinsic protein function of *PtrPIP2:4*. These findings establish a functional link between *PIP2* and adventitious root emergence, elongation, and lateral root formation in woody species, and provides new insights into the understanding of adventitious root emergence, elongation, and lateral root formation.

## 4. Materials and Methods

### 4.1. Plant Material and Growth Conditions

*Populus trichocarpa* (Nisqually-1) was used as the plant material in this study. Wild-type and transgenic plants were propagated on rooting medium consisting of Woody Plant Medium (WPM) supplemented with 25 g·L^−1^ sucrose, and 0.1 mg·L^−1^ indole-3-butyric acid (IBA), with the pH adjusted to 5.8. Culture conditions were maintained at a light intensity of 90 μmol·m^−2^·s^−1^, a temperature of 23–25 °C, and a photoperiod of 16 h light/8 h dark. For gene expression analysis, samples were collected from different tissues of four-month-old wild-type plants, including mature leaves, young leaves, roots, xylem, phloem, and vascular cambium. All samples were immediately frozen in liquid nitrogen and stored at −80 °C until further use.

### 4.2. Identification of P. trichocarpa PIPs

To identify plasma membrane intrinsic protein (PIP) genes in *Populus trichocarpa*, the genome sequences were retrieved from the Phytozome v14 database (https://phytozome-next.jgi.doe.gov/, accessed on 12 April 2025 *Populus trichocarpa* v4.1). A combination of hidden Markov model (HMM) searches with the Pfam domain PF00230 (aquaporin) and BLASTP (https://phytozome-next.jgi.doe.gov/blast-search; accessed on 12 April 2025 *Populus trichocarpa* v4.1) searches using known *Arabidopsis thaliana* PIP sequences as queries was employed [44]. Only sequences containing the conserved aquaporin domain were retained. The identified *PtrPIP* genes were further confirmed by manual curation.

### 4.3. Bioinformatics Analysis

A phylogenetic tree was built by the whole protein sequences of the PtrPIP gene family members and their orthologous from *P. trichocarpa* and *Arabidopsis thaliana* to investigate the relationships of PtrPIPs in *P. trichocarpa*. *A. thaliana*’s *PIP* genes were obtained using an online database (https://www.arabidopsis.org/, accessed on 19 April 2024). Multiple sequence alignment of PtrPIP proteins was performed using ClustalW and the online tool Clustal Omega. Subsequently, a phylogenetic tree was built in MEGA 6.0 via the Neighbor-Joining (NJ) method with 1000 bootstrap replicates. Finally, the chromosomal distribution of the 13 PtrPIP genes was visualized using TBtools-II. Five tools-Plant-mPLoc, LocTree3, WoLF PSORT, YLoc and ngLOC-were used to predict the protein subcellular locations, and the final results were presented according to the majority.

### 4.4. Vector Construction

For the *PtrPIP2:4* overexpression construct, the full-length coding sequence (CDS) of *PtrPIP2:4* was amplified from *P. trichocarpa* cDNA. The CDS of *PtrPIP2:4* was then ligated into the pCAMBIA1300 vector, which had been double-digested with BamH I and Sac I, under the control of the CaMV 35S promoter. The resulting *PtrPIP2:4*-pCAMBIA1300 construct was verified by Sanger sequencing (Shenggong, Shanghai, China) prior to transformation.

### 4.5. RNA Extraction and RT-qPCR Analysis

Total RNA was extracted from the root form of the three independent 15-day-old *PtrPIP2:4*-OE plants (OE3, OE4, and OE7) and WT, using the RNeasy Plant Mini Kit (Qiagen, Duesseldorf, Germany, Cat. No. 74904). First-strand cDNA synthesis was carried out with HIScript QRT SuperMix for qPCR gDNA wiper Kit (Vazyme, Nanjing, China) following the protocol provided by the manufacturer. RT-qPCR was performed using TransStart^®^ Top Green qPCR SuperMix (TransGen Biotech, Beijing, China). qTOWER 3G Cycler was used to work program. The relative expression levels of genes were calculated using the 2^−ΔΔCt^ method. The *PtrActin2* served as the internal control [45,46,47]. Each sample was analyzed with three biological replicates and three technical replicates.

### 4.6. Genetic Transformation of P. trichocarpa

Agrobacterium-mediated transformation of *P. trichocarpa* was performed according to the protocol described by Li et al. [48] and Xu et al. [49]. The overexpressing transgenic plants were identified by RT-qPCR to determine the expression of the transgenes in the entire seedling.

### 4.7. Subcellular Localization

To determine the subcellular localization of PtrPIP2:4, the specific coding sequence of PtrPIP2:4 (excluding the stop codon) was amplified using gene-specific primers containing Spe I homologous sequences. The amplified fragment was then ligated into the Spe I-linearized pBI121-GFP vector using infusion homologous recombination enzyme. The resulting PtrPIP2:4-pBI121-GFP construct was verified by Sanger sequencing (Shenggong, Shanghai) prior to transformation. Plasmids were extracted using the cesium chloride density-gradient ultracentrifugation method [50] and transfected into leaf protoplasts of *Populus trichocarpa*. After 12 h of culture, the transformed protoplasts were observed for GFP signals at 488 nm using confocal laser scanning microscopy (LSM700, Carl Zeiss AG, Oberkochen, Germany).

### 4.8. Analysis of Adventitious Root Formation and Growth

To evaluate the effect of *PtrPIP2:4* overexpression on root development, stem cuttings from WT *P. trichocarpa* and three independent *PtrPIP2:4* overexpression lines (OE3, OE4, and OE7) were used. The basal ends of the cuttings were placed on Woody Plant Medium (WPM) and cultured under identical conditions. The time of adventitious root emergence was recorded daily. After 30 days of culture, the following parameters were measured for each cutting: the number of adventitious roots, the length of adventitious roots (measured using a ruler or image analysis software), and the number of lateral roots per adventitious root. All experiments were performed with at least three biological replicates.

### 4.9. Transcriptome Sequencing

Samples of root were collected from three 15-day-old *PtrPIP2:4*-OE7 plants and WT. Three biological replicates were performed, with each replicate consisting of a pooled sample from three independent plants. Total RNA was extracted using a plant RNA extraction reagent (Bio-Flux, Hangzhou, China), and were sent to the Annoroad Gene Biotechnology Co., Ltd. (Beijing, China) for quality examination, and those that qualified were used for library preparation using the NEB Next^®^ Ultra™ RNA Library Prep Kit (NEB, Ipswich, MA USA). The libraries were sequenced on an Illumina HiSeq 4000 sequencing platform (San Diego, CA, USA). The raw reads were filtered to generate clean reads and mapped to the *P. trichocarpa* genome using HISAT2 version2.1.0 [51]. Read counts for each gene were generated byHTSeq version 0.6.0, and the fragments per kilobase of transcript per million mapped reads (FPKM) were calculated to estimate the expression level of genes in each sample [52]. The data were submitted to the NCBI Sequence Read Archive (accession numbers SRR14040098-SRR14040103).

The transcriptome data processing was performed as previously described [53]. Differential expression analysis between the *PtrPIP2:4* overexpression plants and WT plants was performed using DESeq2 version 1.6.3. The *p*-value was adjusted using Benjamini and Hochberg’s approach for controlling the false discovery rate (FDR). A corrected *p*-value < 0.05 and |log2_ratio| ≥ 1 were set as the threshold for significant differential expression. Kyoto Encyclopedia of Genes and Genomes (KEGG) enrichment analysis of differentially expressed genes (DEGs) was performed using the ClusterProfiler v4.8.3, and a corrected *p*-value of <0.05 was considered significantly enriched among DEGs.

### 4.10. Statistical Analysis

All data analyses and statistical tests were conducted by SPSS version 24.0. Values are displayed as the mean ± standard deviation (SD), and the number of asterisks indicates statistical significance at different levels (* *p* < 0.05, ** *p* < 0.01 and *** *p* < 0.001).

## 5. Conclusions

In this study, we identified *PtrPIP2:4* as a root-specific *PIP2* subtype in *P. trichocarpa* with plasma membrane localization, and demonstrated that its overexpression accelerates adventitious root emergence, promotes root elongation, and increases lateral root number. Combined transcriptomic analysis further revealed that *PtrPIP2:4* may exert its biological functions by modulating the expression of genes involved in regulating the expression of auxin- and flavonoid-related genes. These findings establish a functional link between *PIP2* and adventitious root emergence, elongation, and lateral root formation in woody species, and provides new insights into the understanding of adventitious root emergence, elongation, and lateral root formation.

## Data Availability

All experimental data are provided in the article and Supplementary Materials.

## Supplementary Materials

The following supporting information can be downloaded at: https://www.mdpi.com/article/10.3390/plants15121844/s1, Figure S1: Comparison of the amino acid sequences of 13 *PtrPIPs*. Amino acid sequences are aligned by Cluster W software. Red boxed regions are predicted to form six putative transmembrane-helix (H1 to H6). Black boxed regions refer to the NPA motifs, the most highly conserved amino acid sequences of MIP. The blue dashed area represents an AEF motif, which is conserved in *PtrPIPs*; Figure S2: Chromosomal locations of 13 *PtrPIPs*. Each was mapped to the chromosome based on its physical location. The chromosome number (Chr01-Chr16) displayed on the left. The scale bar represents 5.0 Mb; Table S1: Identification of 13 PtrPIP genes in *P. trichocarpa*; Table S2: Physicochemical properties and predicted localization of the 13 PtPIPs studied here; Table S3: Differential genes in overexpress PtrPIP2:4 plants.
