# Supplementary material for: Overexpression of PtrPIP2:4 Accelerates Adventitious Root Emergence, Promotes Adventitious Root Elongation, and Increases Lateral Root Number in Poplar
Source: Plants (Basel). 2026 Jun 15;15(12):1844. doi: 10.3390/plants15121844 (PMC13306464; doi:10.3390/plants15121844)
Supplement: Supplementary file 1 [file plants-15-01844-s001.zip › Figure S1 and S2.pdf]

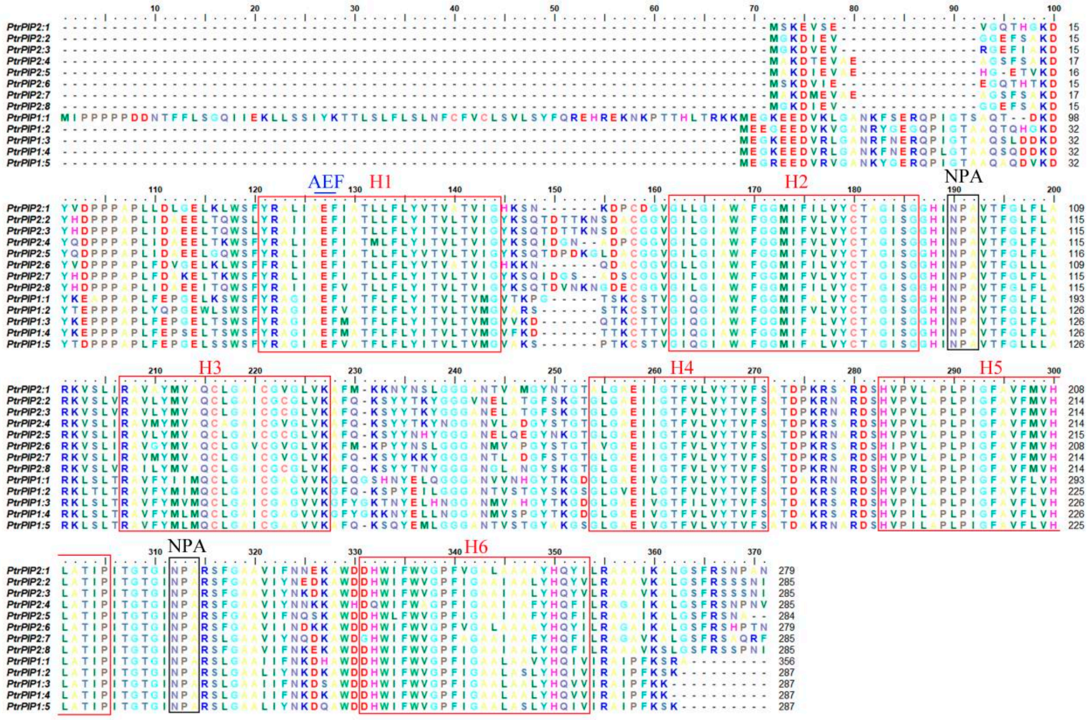

Figure S1. Comparison of the amino acid sequences of 13 *PtrPIPs*. Amino acid sequences are aligned by Cluster W software. Red boxed regions are predicted to form six putative transmembrane-helix (H1 to H6). Black boxed regions refer to the NPA motifs, the most highly conserved amino acid sequences of MIP. The blue dashed area represents an AEF motif, which is conserved in *PtrPIPs.*

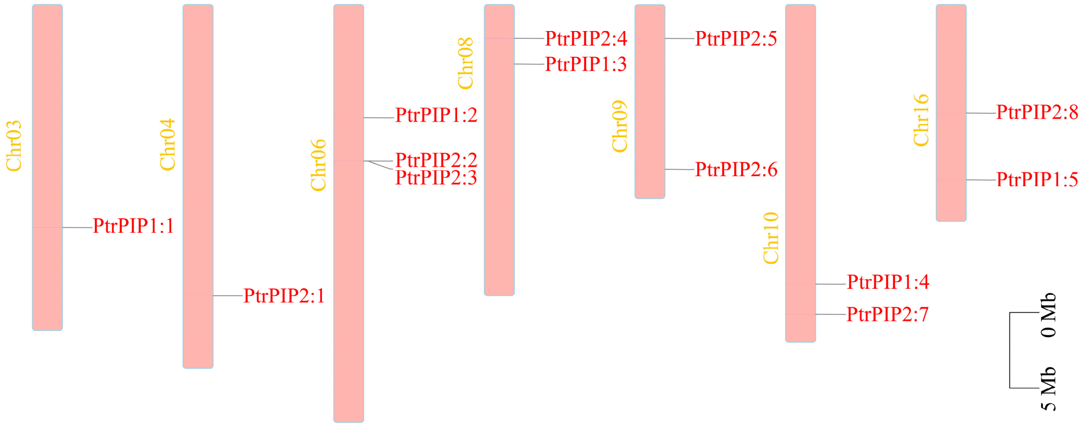

Figure S2. Chromosomal locations of 13 *PtrPIPs*. Each was mapped to the chromosome based on its physical location. The chromosome number (Chr01-Chr16) displayed on the left. The scale bar represents 5.0 Mb.
